# The varied structures of cobalt(II)–pyridine (py)–sulfate: [Co(SO_4_)(py)_4_]_*n*_, [Co_2_(SO_4_)_2_(py)_6_]_*n*_, and [Co_3_(SO_4_)_3_(py)_11_]_*n*_

**DOI:** 10.1107/S205698901901538X

**Published:** 2019-11-19

**Authors:** Ava M. Park, Duyen N. K. Pham, James A. Golen, David R. Manke

**Affiliations:** a Portsmouth Abbey School, 285 Cory’s Lane, Portsmouth, RI, 02871, USA; b University of Massachusetts Dartmouth, 285 Old Westport Rd., North Dartmouth, MA, 02747, USA

**Keywords:** crystal structure, pyridine, sulfate, transition metals, crystal field theory, coordination chemistry, cobalt complexes

## Abstract

The solid-state structures of two cobalt–pyridine–sulfate compounds, namely *catena*-poly[[tetra­kis­(pyridine-κ*N*)cobalt(II)]-μ-sulfato-κ^2^ *O*:*O*′], [Co(SO_4_)(C_5_H_5_N)_4_]_*n*_, (**1**), and *catena*-poly[[tetra­kis­(pyridine-κ*N*)cobalt(II)]-μ-sulfato-κ^3^ *O*:*O*′,*O*′′-[bis­(pyridine-κ*N*)cobalt(II)]-μ-sulfato-κ^3^ *O*,*O*′:*O*′′]_*n*_, [Co_2_(SO_4_)_2_(C_5_H_5_N)_6_]_*n*_, (**2**), are reported. Compound (**1**) displays a polymeric structure, with infinite chains of Co^II^ cations adopting octa­hedral N_4_O_2_ coordination environments that involve four pyridine ligands and two bridging sulfate ions. Compound (**2**) is also polymeric with infinite chains of Co^II^ cations. The first Co center has an octa­hedral N_4_O_2_ coordination environment that involves four pyridine ligands and two bridging sulfate ligands. The second Co center has an octa­hedral N_2_O_4_ coordination environment that involves two pyridine ligands and two bridging sulfate ions that chelate the Co atom. The structure of (**2**) was refined as a two-component inversion twin.

## Abstract 2

The crystal structures of two new forms of cobalt–pyridine–sulfate complexes are presented. The feature infinite chains of metal–pyridine units connected by bridging sulfate anions, which are distinct from the only previously reported structure of a cobalt–pyridine–sulfate compound.

## Chemical context   

The synthesis of metal–pyrdine–sulfates has been reported since the 19th century, when Jørgensen’s chain theory was still the prevailing hypothesis (Reitzenstein, 1894 ▸; Howe, 1898 ▸). Since that time, the structural understanding of metal complexes has greatly increased, first with the acceptance of Werner’s coordination theory (Werner, 1893 ▸), with crystal field theory from Bethe in 1929 (Bethe, 1929 ▸), and the modifications of theory in the ninety years since. Despite the long history of these compounds, their crystallographic study is rather limited. Before we began a crystallographic examination of metal–pyridine–sulfates in 2018, there were only two examples of such complexes without other ligands or components reported in the literature (Cotton & Reid, 1984 ▸; Memon *et al.*, 2006 ▸).

Since we began studying the structural chemistry of metal–pyridine–sulfates, we have observed many different structural motifs in the complexes. The coordination environment of each compound can usually be predicted with crystal field theory, although the exact nature is dependent upon the number of pyridines bound and the binding mode of the sulfate anion. The sulfate anion can have a number of different coordination modes, including μ-sulfato-κ^2^-*O*:*O*, μ-sulfato-κ^2^-*O*:*O*′ and μ-sulfato-κ^3^-*O*:*O*′:*O*". Herein we report two new structures of cobalt–pyridine–sulfates formed by altering the growth conditions and compare these structures with the previously reported structure of a cobalt–pyridine–sulfate and the structures of related complexes.
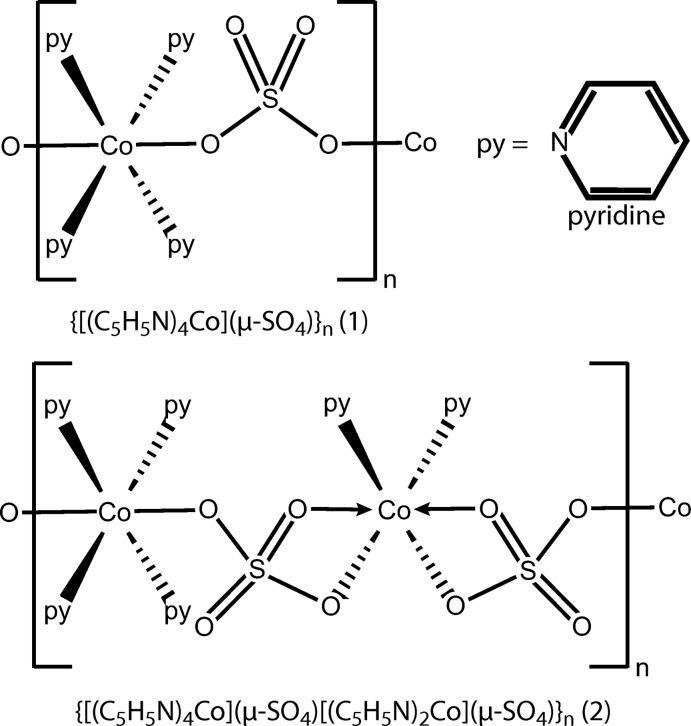


## Structural commentary   

**Figure 1:**
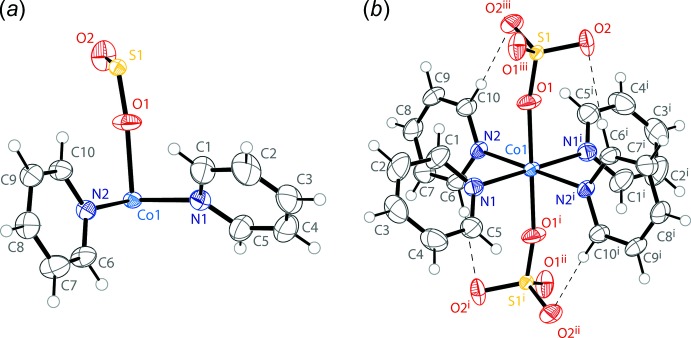
The mol­ecular structure of compound (**1**), including (*a*) the asymmetric unit and (*b*) the coordination environment around Co1. Displacement ellipsoids are drawn at the 50% probability level. H atoms are drawn as spheres of arbitrary radius. C—H⋯O inter­actions (Table 1 ▸) are shown as dashed lines. [Symmetry codes: (i) 1 − *x*, 1 − *y*, 1 − *z*; (ii) *x*, 1 − *y*, −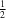 + *z*; (iii) 1 − *x*, *y*, 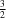 − *z*].

The asymmetric unit of the pink crystals of (**1**) consists of two pyridine mol­ecules and one half of a sulfate anion coordinated to a cobalt atom sitting on an inversion center (Fig. 1 ▸ *a*). When grown out, the cobalt ion shows an octa­hedral coordination environment (Fig. 1 ▸ *b*). The equatorial positions of the octa­hedron are occupied by four pyridine ligands in a square-planar arrangement. The CoN_4_ unit exhibits planarity enforced by symmetry, with *cis* N—Co—N angles of 86.45 (6) and 93.55 (6)°. To complete the octa­hedron, the axial positions are occupied by two sulfate ions, with an inversion enforced O—Co—O angle of 180° and *cis* O—Co—N angles of 88.87 (6) and 91.67 (6)°. The pyridine rings are rotated from the CoN_4_ plane by dihedral angles of 47.30 (10) and 78.33 (9)°. The 78.33 (9)° angles are constrained by two C—H⋯O inter­actions between the *ortho* hydrogen atoms and the two *trans* sulfates (Table 1 ▸).

**Figure 2:**
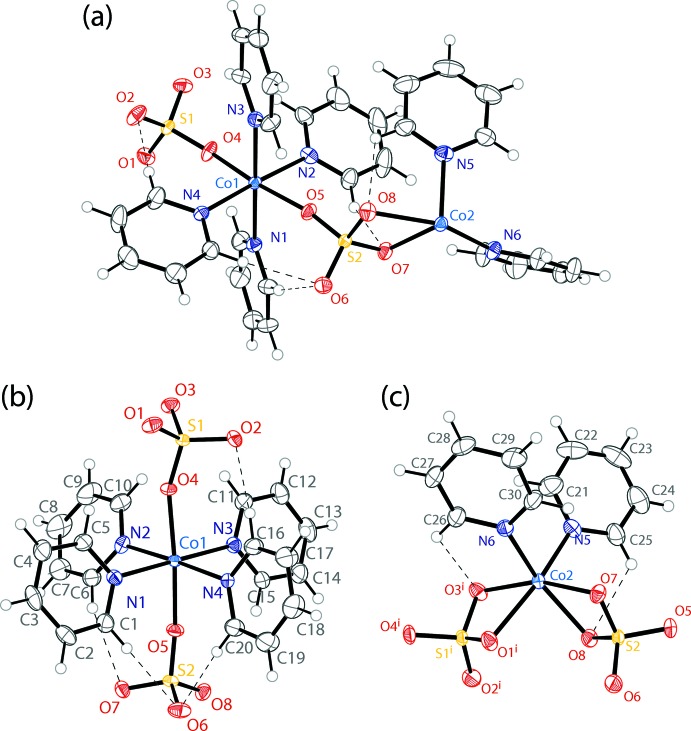
The mol­ecular structure of compound (**2**), including (*a*) the asymmetric unit, (*b*) the coordination environment around Co1, and (*c*) the coordination environment around Co2. Displacement ellipsoids are drawn at the 50% probability level. H atoms are drawn as spheres of arbitrary radius. C—H⋯O inter­actions (Table 2 ▸) are shown as dashed lines. [Symmetry code: (i) −1 + *x*, −1 + *y*, −1 + *z*].

The asymmetric unit of the purple crystals of (**2**) consists of two cobalt atoms, six coordinated pyridines and two sulfate anions (Fig. 2 ▸ *a*). There are two crystallographically unique cobalt atoms, with Co1 (Fig. 2 ▸ *b*) displaying an octa­hedral N_4_O_2_ coordination environment and Co2 (Fig. 2 ▸ *c*) exhibiting an octa­hedral N_2_O_4_ coordination geometry.

Co1 has four pyridine ligands occupying the equatorial positions of an octa­hedron, with the CoN_4_ plane showing a maximum deviation from planarity of 0.018 Å. Two sulfate anions occupy the axial positions to complete the octa­hedral coordination. The *cis* N—Co—N angles have values ranging from 87.48 (13) to 93.18 (12)°, and the *trans* O—Co—O angle is 173.43 (12)°. The planes of the four pyridine rings are rotated from the equatorial CoN_4_ plane by dihedral angles of 58.6 (2), 64.6 (2), 65.6 (2), and 73.1 (2)°. Two of the rings show one C—H⋯O inter­action with an *ortho* hydrogen atom, one ring shows two C—H⋯O inter­actions with two *ortho* hydrogen atoms, and the fourth ring shows no C—H⋯O inter­actions (Table 2 ▸).

Co2 is bound by two pyridine ligands and two chelating sulfate anions to give an octa­hedral coordination environment. The pyridine rings adopt a *cis* configuration, with an N—Co—N angle of 93.63 (13)°. The two sulfate ligands exhibit O—Co—O bite angles of 65.90 (10) and 66.37 (10)°. The other *cis* O—Co—O angles are 86.87 (11), 98.98 (11), and 102.84 (11)°, and the six *cis* N—Co—O angles range from 92.49 (12) to 98.33 (13)°. Each pyridine ring is involved in *ortho* C—H⋯O inter­actions (Table 2 ▸).

## Supra­molecular features   

**Figure 3:**
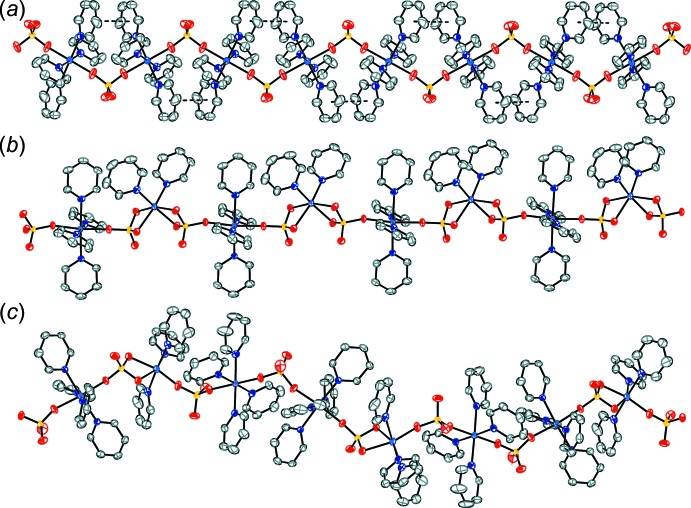
The infinite chains of (*a*) compound (**1**) along [001], (*b*) compound (**2**) along [111], and (*c*) the previously reported cobalt–pyridine-sulfate complex [Co_3_(SO_4_)_3_(C_5_H_5_N)_11_]_*n*_ along [001] (Pham *et al.*, 2019 ▸). Displacement ellipsoids are drawn at the 50% probability level. H atoms are omitted for clarity. The π–π inter­actions in (**1**) are shown as dashed lines.

The Co^II^ atoms in compound (**1**) are linked together into infinite chains along the [001] direction through sulfate anions with O—S—O bridges (Figs. 3 ▸ *a*, 4 ▸ *a*). Between each successive tetra­pyridine cobalt unit, there are parallel slipped π–π inter­actions [inter-centroid distance: 3.637 (1) Å, inter-planar distance: 3.611 (1) Å, slippage: 0.435 (1) Å].

The Co^II^ atoms in compound (**2**) are linked together into infinite chains along the [111] direction through the sulfate anions (Figs. 3 ▸ *b*, 4 ▸ *b*). The chain alternates between tetra­pyridine cobalt units and di­pyridine cobalt units. No π–π inter­actions are observed in the crystal.

## Database survey   

In a prior publication, we reported the structure of another cobalt–pyridine–sulfate [Co_3_(SO_4_)_3_(C_5_H_5_N)_11_)]_*n*_, which was grown at a lower concentration of cobalt. This structure shows two successive octa­hedral cobalt atoms with N_4_O_2_ coordination, where each atom is coordinated to four pyridines and two bridging sulfates. The third cobalt atom in the chain shows N_3_O_3_ coordination where three pyridines are bound and there are two sulfates bound, one of which is chelating to the cobalt (Pham *et al.*, 2018 ▸). Fig. 3 ▸ compares the chain structure of this complex with those of compounds (**1**) and (**2**). In compound (**1**), every cobalt atom possesses an octa­hedral N_4_O_2_ coord­in­ation. This complex is isostructural with the structure observed for the iron and nickel pyridine–sulfate complexes (Roy *et al.*, 2018 ▸). This structural motif is also consistent with that observed for the 4-picoline–sulfate structures of iron, cobalt, nickel and cadmium (Pham *et al.*, 2019 ▸). In compound (**2**), the cobalt atoms alternate between N_4_O_2_ coordination and N_2_O_4_ coordination. This tetra­pyridine/bi­pyridine alternation is similar to what is observed in the zinc–pyridine–sulfate structure, which alternates between octa­hedral and tetra­hedral zinc centers. In the case of cobalt, the bis­(pyridine) cobalt center is still octa­hedral because the two coordinated sulfates both chelate to the cobalt. The end result is an infinite chain of octa­hedral cobalt atoms, which is true in compound (**1**) and the previously reported cobalt–pyridine–sulfate complex. The methane­sulfato complexes of cobalt (II) have also been reported as octa­hedral tetra­kis­(pyridine), [Co(SO_3_CH_3_)_2_(py)_4_], and octa­hedral bis­(pyridine), [Co(SO_3_CH_3_)_2_(py)_2_], compounds, consistent with the two independent cobalt centers observed in (**2**) (Johnson *et al.*, 1977 ▸).

## Synthesis and crystallization   

For compound (**1**), 40 mg of cobalt sulfate hepta­hydrate (J. T. Baker) was dissolved in pyridine (2 mL, Fischer Chemical) and distilled water (100 µL) in a 20 mL vial. The vial was heated to 338 K for 48 h, after which single crystals suitable for X-ray diffraction studies were isolated from the reaction mixture.

For compound (**2**), 48 mg of cobalt sulfate hepta­hydrate (J. T. Baker) was dissolved in pyridine (2 mL, Fischer Chemical) and distilled water (30 µL) in a 20 mL vial. The vial was heated to 358 K for 48 h, after which single crystals suitable for X-ray diffraction studies were isolated from the reaction mixture.

## Refinement   

Crystal data, data collection and structure refinement details are summarized in Table 3 ▸. All structure solutions were obtained by intrinsic phasing. All non-hydrogen atoms were refined anisotropically (*SHELXL*) by full-matrix least squares on *F* ^2^. Hydrogen atoms were placed in calculated positions and then refined with a riding model with C—H bond lengths of 0.95 Å and with isotropic displacement parameters set to 1.20 *U*_eq_ of the parent C atom. The structre of (**2**) was refined as a two-component inversion twin, BASF = 0.165 (13).

## Supplementary Material

Crystal structure: contains datablock(s) 1, 2, I. DOI: 10.1107/S205698901901538X/sj5586sup1.cif

Structure factors: contains datablock(s) 1. DOI: 10.1107/S205698901901538X/sj55861sup2.hkl

Structure factors: contains datablock(s) 2. DOI: 10.1107/S205698901901538X/sj55862sup3.hkl

CCDC references: 1965662, 1965663, 1965662, 1965663

Additional supporting information:  crystallographic information; 3D view; checkCIF report

## Figures and Tables

**Figure 4:**
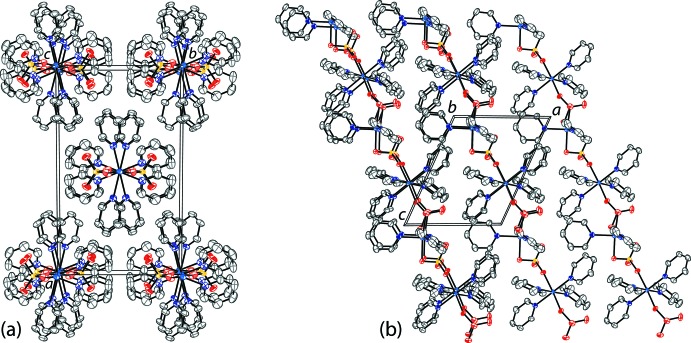
The packing of (*a*) compound (**1**) along the *c*-axis and (*b*) compound (**2**) along the *b*-axis. Displacement ellipsoids are drawn at the 50% probability level. H atoms are omitted for clarity.

**Table 1:** Hydrogen-bond geometry (Å, °) for (**1**)

*D*—H⋯*A*	*D*—H	H⋯*A*	*D*⋯*A*	*D*—H⋯*A*
C6—H6*A*⋯O1^i^	0.93	2.51	3.106 (2)	122
C6—H6*A*⋯O2^i^	0.93	2.51	3.429 (3)	171
C10—H10*A*⋯O1	0.93	2.48	3.046 (2)	120
C10—H10*A*⋯O2^ii^	0.93	2.43	3.353 (3)	171

Symmetry codes: (i) 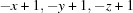; (ii) 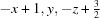.

**Table 2:** Hydrogen-bond geometry (Å, °) for (**2**)

*D*—H⋯*A*	*D*—H	H⋯*A*	*D*⋯*A*	*D*—H⋯*A*
C1—H1*A*⋯O6	0.95	2.63	3.563 (6)	167
C2—H2*A*⋯O2^i^	0.95	2.52	3.219 (5)	131
C5—H5*A*⋯O4	0.95	2.42	3.009 (5)	120
C6—H6*A*⋯O5	0.95	2.56	3.054 (5)	112
C6—H6*A*⋯O7	0.95	2.47	3.322 (6)	149
C10—H10*A*⋯O4	0.95	2.53	3.010 (5)	112
C12—H12*A*⋯O7^ii^	0.95	2.60	3.271 (5)	128
C15—H15*A*⋯O5	0.95	2.45	3.017 (5)	118
C16—H16*A*⋯O2	0.95	2.19	3.139 (6)	176
C20—H20*A*⋯O5	0.95	2.51	3.091 (5)	119
C20—H20*A*⋯O6	0.95	2.32	3.272 (5)	175
C25—H25*A*⋯O8	0.95	2.55	3.162 (5)	123
C30—H30*A*⋯O7	0.95	2.54	3.116 (5)	119

Symmetry codes: (i) 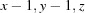; (ii) 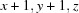.

**Table 3:** Experimental details

	(**1**)	(**2**)
Crystal data		
Chemical formula	[Co(SO_4_)(C_5_H_5_N)_4_]	[Co_2_(SO_4_)_2_(C_5_H_5_N)_6_]
*M*_r_	471.39	784.58
Crystal system, space group	Monoclinic, *C*2/*c*	Triclinic, *P*1
Temperature (K)	295	200
*a*, *b*, *c* (Å)	18.6323 (18), 10.0803 (9), 11.9403 (11)	9.5795 (6), 9.7612 (5), 10.7219 (6)
α, β, γ (°)	90, 115.945 (3), 90	98.488 (2), 107.697 (2), 115.948 (2)
*V* (Å^3^)	2016.6 (3)	811.46 (8)
*Z*	4	1
Radiation type	Mo *K*α	Mo *K*α
μ (mm^−1^)	0.99	1.21
Crystal size (mm)	0.28 × 0.13 × 0.06	0.25 × 0.20 × 0.02

Data collection		
Diffractometer	Bruker APEXIII CMOS	Bruker APEXIII photon2
Absorption correction	Multi-scan (*SADABS*; Bruker, 2016 ▸)	Multi-scan (*SADABS*; Bruker, 2016 ▸)
*T*_min_, *T*_max_	0.667, 0.745	0.661, 0.745
No. of measured, independent and observed [*I* > 2σ(*I*)] reflections	20212, 1854, 1572	22679, 6013, 5906
*R*_int_	0.071	0.026
(sin θ/λ)_max_ (Å^−1^)	0.604	0.610

Refinement		
*R*[*F* ^2^ > 2σ(*F* ^2^)], *wR*(*F* ^2^), *S*	0.027, 0.063, 1.02	0.026, 0.072, 1.03
No. of reflections	1854	6013
No. of parameters	139	434
No. of restraints	0	3
H-atom treatment	H-atom parameters constrained	H-atom parameters constrained
Δρ_max_, Δρ_min_ (e Å^−3^)	0.24, −0.24	0.79, −0.30
Absolute structure	–	Refined as an inversion twin
Absolute structure parameter	–	0.165 (13)

Computer programs: *APEX3* and *SAINT* (Bruker, 2016 ▸), *SHELXT* (Sheldrick, 2015*a* ▸), *SHELXL2014* (Sheldrick, 2015*b* ▸), *OLEX2* (Dolomanov *et al.*, 2009 ▸), *SHELXTL* (Sheldrick, 2008 ▸), and *publCIF* (Westrip, 2010 ▸).

## supplementary crystallographic information

### catena-Poly[[tetrakis(pyridine-κN)cobalt(II)]-µ-sulfato-κ2O:O'] (1) Crystal data

**Table:**

[Co(SO_4_)(C_5_H_5_N)_4_]	*F*(000) = 972
*M**_r_* = 471.39	*D*_x_ = 1.553 Mg m^−^^3^
Monoclinic, *C*2/*c*	Mo *K*α radiation, λ = 0.71073 Å
*a* = 18.6323 (18) Å	Cell parameters from 8333 reflections
*b* = 10.0803 (9) Å	θ = 3.3–25.3°
*c* = 11.9403 (11) Å	µ = 0.99 mm^−^^1^
β = 115.945 (3)°	*T* = 295 K
*V* = 2016.6 (3) Å^3^	BLOCK, pink
*Z* = 4	0.28 × 0.13 × 0.06 mm

### catena-Poly[[tetrakis(pyridine-κN)cobalt(II)]-µ-sulfato-κ2O:O'] (1) Data collection

**Table:**

Bruker APEXIII CMOS diffractometer	1572 reflections with *I* > 2σ(*I*)
φ and ω scans	*R*_int_ = 0.071
Absorption correction: multi-scan (SADABS; Bruker, 2016)	θ_max_ = 25.4°, θ_min_ = 3.3°
*T*_min_ = 0.667, *T*_max_ = 0.745	*h* = −22→22
20212 measured reflections	*k* = −12→12
1854 independent reflections	*l* = −14→14

### catena-Poly[[tetrakis(pyridine-κN)cobalt(II)]-µ-sulfato-κ2O:O'] (1) Refinement

**Table:**

Refinement on *F*^2^	Hydrogen site location: inferred from neighbouring sites
Least-squares matrix: full	H-atom parameters constrained
*R*[*F*^2^ > 2σ(*F*^2^)] = 0.027	*w* = 1/[σ^2^(*F*_o_^2^) + (0.0254*P*)^2^ + 2.4084*P*] where *P* = (*F*_o_^2^ + 2*F*_c_^2^)/3
*wR*(*F*^2^) = 0.063	(Δ/σ)_max_ < 0.001
*S* = 1.02	Δρ_max_ = 0.24 e Å^−^^3^
1854 reflections	Δρ_min_ = −0.24 e Å^−^^3^
139 parameters	Extinction correction: SHELXL, Fc^*^=kFc[1+0.001xFc^2^λ^3^/sin(2θ)]^-1/4^
0 restraints	Extinction coefficient: 0.0019 (3)

### catena-Poly[[tetrakis(pyridine-κN)cobalt(II)]-µ-sulfato-κ2O:O'] (1) Special details

**Table:**

Geometry. All esds (except the esd in the dihedral angle between two l.s. planes) are estimated using the full covariance matrix. The cell esds are taken into account individually in the estimation of esds in distances, angles and torsion angles; correlations between esds in cell parameters are only used when they are defined by crystal symmetry. An approximate (isotropic) treatment of cell esds is used for estimating esds involving l.s. planes.

### catena-Poly[[tetrakis(pyridine-κN)cobalt(II)]-µ-sulfato-κ2O:O'] (1) Fractional atomic coordinates and isotropic or equivalent isotropic displacement parameters (Å^2^)

**Table:**

	*x*	*y*	*z*	*U*_iso_*/*U*_eq_	
Co1	0.5000	0.5000	0.5000	0.02070 (13)	
O1	0.49333 (8)	0.59836 (16)	0.64714 (13)	0.0406 (4)	
O2	0.57186 (9)	0.76549 (16)	0.79112 (15)	0.0433 (4)	
N1	0.45647 (10)	0.32138 (17)	0.55318 (15)	0.0305 (4)	
N2	0.37320 (9)	0.55056 (17)	0.38523 (14)	0.0257 (4)	
C1	0.39850 (16)	0.3286 (3)	0.5901 (2)	0.0503 (7)	
H1A	0.3780	0.4116	0.5942	0.060*	
C2	0.36775 (18)	0.2191 (3)	0.6224 (3)	0.0649 (8)	
H2A	0.3265	0.2285	0.6457	0.078*	
C3	0.39806 (18)	0.0971 (3)	0.6200 (2)	0.0594 (8)	
H3A	0.3780	0.0216	0.6410	0.071*	
C4	0.45897 (19)	0.0885 (3)	0.5858 (3)	0.0582 (7)	
H4A	0.4823	0.0069	0.5857	0.070*	
C5	0.48541 (15)	0.2016 (2)	0.5514 (2)	0.0448 (6)	
H5A	0.5257	0.1937	0.5257	0.054*	
C6	0.33165 (12)	0.4887 (2)	0.2775 (2)	0.0360 (5)	
H6A	0.3577	0.4259	0.2513	0.043*	
C7	0.25194 (13)	0.5129 (3)	0.2028 (2)	0.0469 (6)	
H7A	0.2255	0.4687	0.1274	0.056*	
C8	0.21197 (12)	0.6030 (2)	0.2410 (2)	0.0404 (6)	
H8A	0.1584	0.6218	0.1919	0.048*	
C9	0.25326 (12)	0.6644 (2)	0.3534 (2)	0.0370 (5)	
H9A	0.2276	0.7240	0.3832	0.044*	
C10	0.33304 (12)	0.6372 (2)	0.42193 (19)	0.0331 (5)	
H10A	0.3605	0.6809	0.4974	0.040*	
S1	0.5000	0.68569 (6)	0.7500	0.01886 (16)	

### catena-Poly[[tetrakis(pyridine-κN)cobalt(II)]-µ-sulfato-κ2O:O'] (1) Atomic displacement parameters (Å^2^)

**Table:**

	*U*^11^	*U*^22^	*U*^33^	*U*^12^	*U*^13^	*U*^23^
Co1	0.0187 (2)	0.0246 (2)	0.0198 (2)	0.00353 (15)	0.00935 (15)	−0.00111 (15)
O1	0.0358 (8)	0.0582 (11)	0.0268 (8)	0.0111 (8)	0.0128 (6)	−0.0138 (7)
O2	0.0303 (8)	0.0391 (9)	0.0562 (10)	−0.0150 (7)	0.0148 (7)	0.0026 (8)
N1	0.0321 (9)	0.0314 (10)	0.0290 (9)	0.0009 (8)	0.0141 (8)	0.0004 (8)
N2	0.0212 (8)	0.0300 (9)	0.0261 (9)	0.0040 (7)	0.0105 (7)	0.0013 (7)
C1	0.0583 (16)	0.0482 (16)	0.0638 (16)	0.0080 (13)	0.0445 (14)	0.0116 (13)
C2	0.0691 (19)	0.070 (2)	0.079 (2)	−0.0043 (16)	0.0536 (17)	0.0158 (16)
C3	0.083 (2)	0.0493 (17)	0.0534 (16)	−0.0231 (15)	0.0364 (15)	0.0029 (13)
C4	0.085 (2)	0.0324 (14)	0.0665 (18)	−0.0050 (13)	0.0420 (16)	−0.0024 (13)
C5	0.0510 (14)	0.0351 (13)	0.0563 (15)	−0.0006 (11)	0.0309 (12)	0.0000 (11)
C6	0.0268 (11)	0.0426 (13)	0.0359 (11)	0.0071 (10)	0.0114 (9)	−0.0080 (10)
C7	0.0286 (12)	0.0580 (16)	0.0401 (13)	0.0042 (11)	0.0021 (10)	−0.0160 (12)
C8	0.0214 (11)	0.0496 (14)	0.0440 (13)	0.0096 (10)	0.0085 (10)	0.0030 (11)
C9	0.0304 (11)	0.0438 (14)	0.0399 (12)	0.0135 (10)	0.0183 (10)	0.0025 (10)
C10	0.0282 (11)	0.0406 (12)	0.0289 (11)	0.0058 (9)	0.0110 (9)	−0.0033 (9)
S1	0.0181 (3)	0.0204 (3)	0.0206 (3)	0.000	0.0108 (3)	0.000

### catena-Poly[[tetrakis(pyridine-κN)cobalt(II)]-µ-sulfato-κ2O:O'] (1) Geometric parameters (Å, º)

**Table:**

Co1—O1^i^	2.0679 (13)	C3—C4	1.367 (4)
Co1—O1	2.0679 (13)	C3—H3A	0.9300
Co1—N1	2.1803 (17)	C4—C5	1.373 (3)
Co1—N1^i^	2.1803 (17)	C4—H4A	0.9300
Co1—N2	2.2105 (15)	C5—H5A	0.9300
Co1—N2^i^	2.2105 (15)	C6—C7	1.379 (3)
O1—S1	1.4715 (14)	C6—H6A	0.9300
O2—S1	1.4511 (14)	C7—C8	1.373 (3)
N1—C5	1.327 (3)	C7—H7A	0.9300
N1—C1	1.335 (3)	C8—C9	1.368 (3)
N2—C6	1.331 (3)	C8—H8A	0.9300
N2—C10	1.342 (2)	C9—C10	1.375 (3)
C1—C2	1.373 (4)	C9—H9A	0.9300
C1—H1A	0.9300	C10—H10A	0.9300
C2—C3	1.359 (4)	S1—O2^ii^	1.4511 (14)
C2—H2A	0.9300	S1—O1^ii^	1.4715 (14)
			
O1^i^—Co1—O1	180.0	C2—C3—H3A	121.0
O1^i^—Co1—N1	91.13 (6)	C4—C3—H3A	121.0
O1—Co1—N1	88.87 (6)	C3—C4—C5	119.3 (3)
O1^i^—Co1—N1^i^	88.87 (6)	C3—C4—H4A	120.3
O1—Co1—N1^i^	91.13 (6)	C5—C4—H4A	120.3
N1—Co1—N1^i^	180.0	N1—C5—C4	123.3 (2)
O1^i^—Co1—N2	91.66 (6)	N1—C5—H5A	118.3
O1—Co1—N2	88.34 (6)	C4—C5—H5A	118.3
N1—Co1—N2	86.45 (6)	N2—C6—C7	123.24 (19)
N1^i^—Co1—N2	93.55 (6)	N2—C6—H6A	118.4
O1^i^—Co1—N2^i^	88.33 (6)	C7—C6—H6A	118.4
O1—Co1—N2^i^	91.67 (6)	C8—C7—C6	119.3 (2)
N1—Co1—N2^i^	93.55 (6)	C8—C7—H7A	120.3
N1^i^—Co1—N2^i^	86.45 (6)	C6—C7—H7A	120.3
N2—Co1—N2^i^	180.0	C9—C8—C7	118.1 (2)
S1—O1—Co1	168.95 (11)	C9—C8—H8A	120.9
C5—N1—C1	116.6 (2)	C7—C8—H8A	120.9
C5—N1—Co1	122.80 (14)	C8—C9—C10	119.39 (19)
C1—N1—Co1	120.61 (16)	C8—C9—H9A	120.3
C6—N2—C10	116.66 (17)	C10—C9—H9A	120.3
C6—N2—Co1	120.20 (13)	N2—C10—C9	123.22 (19)
C10—N2—Co1	123.03 (13)	N2—C10—H10A	118.4
N1—C1—C2	123.1 (2)	C9—C10—H10A	118.4
N1—C1—H1A	118.5	O2—S1—O2^ii^	112.67 (14)
C2—C1—H1A	118.5	O2—S1—O1	110.03 (9)
C3—C2—C1	119.5 (2)	O2^ii^—S1—O1	108.71 (8)
C3—C2—H2A	120.2	O2—S1—O1^ii^	108.71 (8)
C1—C2—H2A	120.2	O2^ii^—S1—O1^ii^	110.03 (9)
C2—C3—C4	118.1 (2)	O1—S1—O1^ii^	106.52 (14)
			
C5—N1—C1—C2	−1.5 (4)	N2—C6—C7—C8	−1.4 (4)
Co1—N1—C1—C2	178.7 (2)	C6—C7—C8—C9	−0.7 (4)
N1—C1—C2—C3	1.5 (5)	C7—C8—C9—C10	1.9 (3)
C1—C2—C3—C4	0.4 (5)	C6—N2—C10—C9	−0.8 (3)
C2—C3—C4—C5	−2.1 (4)	Co1—N2—C10—C9	−176.96 (16)
C1—N1—C5—C4	−0.3 (4)	C8—C9—C10—N2	−1.2 (3)
Co1—N1—C5—C4	179.4 (2)	Co1—O1—S1—O2	−16.8 (5)
C3—C4—C5—N1	2.2 (4)	Co1—O1—S1—O2^ii^	107.0 (5)
C10—N2—C6—C7	2.1 (3)	Co1—O1—S1—O1^ii^	−134.5 (5)
Co1—N2—C6—C7	178.36 (19)		

Symmetry codes: (i) −*x*+1, −*y*+1, −*z*+1; (ii) −*x*+1, *y*, −*z*+3/2.

### catena-Poly[[tetrakis(pyridine-κN)cobalt(II)]-µ-sulfato-κ2O:O'] (1) Hydrogen-bond geometry (Å, º)

**Table:**

*D*—H···*A*	*D*—H	H···*A*	*D*···*A*	*D*—H···*A*
C6—H6*A*···O1^i^	0.93	2.51	3.106 (2)	122
C6—H6*A*···O2^i^	0.93	2.51	3.429 (3)	171
C10—H10*A*···O1	0.93	2.48	3.046 (2)	120
C10—H10*A*···O2^ii^	0.93	2.43	3.353 (3)	171

Symmetry codes: (i) −*x*+1, −*y*+1, −*z*+1; (ii) −*x*+1, *y*, −*z*+3/2.

### (2) Crystal data

**Table:**

[Co(SO_4_)_2_(C_5_H_6_N)_6_]	*Z* = 1
*M**_r_* = 784.58	*F*(000) = 402
Triclinic, *P*1	*D*_x_ = 1.606 Mg m^−^^3^
*a* = 9.5795 (6) Å	Mo *K*α radiation, λ = 0.71073 Å
*b* = 9.7612 (5) Å	Cell parameters from 9920 reflections
*c* = 10.7219 (6) Å	θ = 2.6–25.7°
α = 98.488 (2)°	µ = 1.21 mm^−^^1^
β = 107.697 (2)°	*T* = 200 K
γ = 115.948 (2)°	PLATE, purple
*V* = 811.46 (8) Å^3^	0.25 × 0.20 × 0.02 mm

### (2) Data collection

**Table:**

Bruker APEXIII photon2 diffractometer	5906 reflections with *I* > 2σ(*I*)
φ and ω scans	*R*_int_ = 0.026
Absorption correction: multi-scan (SADABS; Bruker, 2016)	θ_max_ = 25.7°, θ_min_ = 3.1°
*T*_min_ = 0.661, *T*_max_ = 0.745	*h* = −11→11
22679 measured reflections	*k* = −11→11
6013 independent reflections	*l* = −13→13

### (2) Refinement

**Table:**

Refinement on *F*^2^	Hydrogen site location: inferred from neighbouring sites
Least-squares matrix: full	H-atom parameters constrained
*R*[*F*^2^ > 2σ(*F*^2^)] = 0.026	*w* = 1/[σ^2^(*F*_o_^2^) + (0.0524*P*)^2^ + 0.1142*P*] where *P* = (*F*_o_^2^ + 2*F*_c_^2^)/3
*wR*(*F*^2^) = 0.072	(Δ/σ)_max_ < 0.001
*S* = 1.03	Δρ_max_ = 0.79 e Å^−^^3^
6013 reflections	Δρ_min_ = −0.29 e Å^−^^3^
434 parameters	Absolute structure: Refined as an inversion twin
3 restraints	Absolute structure parameter: 0.165 (13)

### (2) Special details

**Table:**

Geometry. All esds (except the esd in the dihedral angle between two l.s. planes) are estimated using the full covariance matrix. The cell esds are taken into account individually in the estimation of esds in distances, angles and torsion angles; correlations between esds in cell parameters are only used when they are defined by crystal symmetry. An approximate (isotropic) treatment of cell esds is used for estimating esds involving l.s. planes.
Refinement. Refined as a 2-component inversion twin. BASF 0.16482

### (2) Fractional atomic coordinates and isotropic or equivalent isotropic displacement parameters (Å^2^)

**Table:**

	*x*	*y*	*z*	*U*_iso_*/*U*_eq_	
Co1	0.84425 (5)	0.55261 (5)	0.61510 (4)	0.01356 (12)	
Co2	0.24785 (6)	0.13866 (6)	0.10725 (5)	0.01665 (13)	
S1	1.17468 (11)	0.89862 (10)	0.89114 (9)	0.01596 (19)	
S2	0.51931 (11)	0.19943 (10)	0.33225 (9)	0.0164 (2)	
O1	1.1869 (4)	0.8903 (3)	1.0308 (3)	0.0257 (6)	
O2	1.3166 (4)	0.9028 (4)	0.8658 (4)	0.0307 (7)	
O3	1.1721 (4)	1.0501 (3)	0.8887 (3)	0.0241 (6)	
O4	1.0118 (3)	0.7597 (3)	0.7855 (3)	0.0244 (6)	
O5	0.6576 (4)	0.3553 (3)	0.4393 (3)	0.0217 (6)	
O6	0.5406 (4)	0.0691 (4)	0.3655 (3)	0.0301 (7)	
O7	0.3509 (3)	0.1777 (3)	0.3224 (3)	0.0223 (6)	
O8	0.5184 (4)	0.2064 (4)	0.1943 (3)	0.0230 (6)	
N1	0.7283 (4)	0.4360 (4)	0.7445 (4)	0.0204 (7)	
N2	0.6741 (4)	0.6487 (4)	0.5820 (4)	0.0215 (7)	
N3	0.9613 (4)	0.6672 (4)	0.4849 (3)	0.0200 (7)	
N4	1.0052 (4)	0.4460 (4)	0.6447 (3)	0.0176 (7)	
N5	0.3110 (5)	0.3784 (4)	0.1165 (4)	0.0246 (7)	
N6	0.0033 (4)	0.0704 (4)	0.0989 (4)	0.0229 (7)	
C1	0.6334 (6)	0.2757 (5)	0.7094 (5)	0.0272 (9)	
H1A	0.6163	0.2109	0.6247	0.033*	
C2	0.5587 (6)	0.1993 (6)	0.7905 (5)	0.0336 (10)	
H2A	0.4927	0.0850	0.7619	0.040*	
C3	0.5820 (6)	0.2929 (7)	0.9138 (5)	0.0369 (11)	
H3A	0.5347	0.2447	0.9728	0.044*	
C4	0.6773 (6)	0.4601 (7)	0.9487 (5)	0.0370 (11)	
H4A	0.6929	0.5281	1.0310	0.044*	
C5	0.7486 (6)	0.5257 (5)	0.8629 (4)	0.0286 (9)	
H5A	0.8152	0.6397	0.8889	0.034*	
C6	0.5027 (6)	0.5482 (6)	0.5237 (5)	0.0306 (10)	
H6A	0.4581	0.4352	0.4991	0.037*	
C7	0.3906 (7)	0.6036 (7)	0.4988 (6)	0.0488 (15)	
H7A	0.2711	0.5296	0.4589	0.059*	
C8	0.4526 (8)	0.7670 (8)	0.5321 (7)	0.0532 (16)	
H8A	0.3771	0.8074	0.5150	0.064*	
C9	0.6240 (8)	0.8687 (7)	0.5899 (6)	0.0464 (14)	
H9A	0.6701	0.9819	0.6137	0.056*	
C10	0.7326 (6)	0.8065 (6)	0.6143 (5)	0.0296 (10)	
H10A	0.8524	0.8793	0.6554	0.036*	
C11	1.0601 (5)	0.8273 (5)	0.5168 (4)	0.0246 (9)	
H11A	1.0821	0.8958	0.6019	0.030*	
C12	1.1313 (6)	0.8974 (6)	0.4317 (5)	0.0316 (10)	
H12A	1.1984	1.0113	0.4572	0.038*	
C13	1.1031 (6)	0.7992 (7)	0.3097 (5)	0.0355 (11)	
H13A	1.1498	0.8437	0.2491	0.043*	
C14	1.0062 (6)	0.6355 (6)	0.2778 (5)	0.0354 (11)	
H14A	0.9873	0.5649	0.1953	0.042*	
C15	0.9365 (5)	0.5741 (5)	0.3658 (5)	0.0277 (9)	
H15A	0.8676	0.4604	0.3410	0.033*	
C16	1.1720 (6)	0.5345 (6)	0.7278 (6)	0.0349 (11)	
H16A	1.2212	0.6471	0.7689	0.042*	
C17	1.2763 (6)	0.4698 (7)	0.7568 (6)	0.0461 (14)	
H17A	1.3939	0.5371	0.8169	0.055*	
C18	1.2079 (7)	0.3067 (7)	0.6978 (6)	0.0444 (13)	
H18A	1.2760	0.2588	0.7180	0.053*	
C19	1.0382 (7)	0.2158 (6)	0.6090 (6)	0.0429 (13)	
H19A	0.9879	0.1040	0.5632	0.051*	
C20	0.9411 (6)	0.2882 (5)	0.5866 (5)	0.0295 (10)	
H20A	0.8230	0.2227	0.5272	0.035*	
C21	0.1897 (7)	0.4092 (6)	0.0510 (6)	0.0390 (11)	
H21A	0.0781	0.3207	−0.0062	0.047*	
C22	0.2193 (8)	0.5635 (7)	0.0626 (7)	0.0458 (13)	
H22A	0.1299	0.5798	0.0139	0.055*	
C23	0.3786 (8)	0.6921 (6)	0.1451 (6)	0.0475 (15)	
H23A	0.4017	0.7994	0.1571	0.057*	
C24	0.5055 (8)	0.6619 (6)	0.2107 (6)	0.0456 (13)	
H24A	0.6186	0.7487	0.2666	0.055*	
C25	0.4666 (6)	0.5049 (5)	0.1944 (5)	0.0337 (10)	
H25A	0.5548	0.4861	0.2408	0.040*	
C26	−0.1383 (6)	−0.0208 (6)	−0.0188 (5)	0.0321 (10)	
H26A	−0.1291	−0.0677	−0.0979	0.039*	
C27	−0.2969 (6)	−0.0497 (6)	−0.0301 (5)	0.0384 (11)	
H27A	−0.3944	−0.1150	−0.1154	0.046*	
C28	−0.3120 (6)	0.0175 (6)	0.0842 (6)	0.0368 (11)	
H28A	−0.4192	0.0019	0.0782	0.044*	
C29	−0.1684 (7)	0.1076 (7)	0.2070 (6)	0.0441 (13)	
H29A	−0.1751	0.1530	0.2882	0.053*	
C30	−0.0132 (6)	0.1308 (6)	0.2096 (5)	0.0338 (10)	
H30A	0.0855	0.1927	0.2945	0.041*	

### (2) Atomic displacement parameters (Å^2^)

**Table:**

	*U*^11^	*U*^22^	*U*^33^	*U*^12^	*U*^13^	*U*^23^
Co1	0.0131 (2)	0.0118 (2)	0.0131 (2)	0.00517 (18)	0.00466 (18)	0.00285 (17)
Co2	0.0152 (2)	0.0145 (2)	0.0156 (2)	0.00629 (19)	0.00422 (19)	0.00207 (18)
S1	0.0135 (4)	0.0128 (4)	0.0152 (4)	0.0041 (3)	0.0040 (3)	0.0009 (3)
S2	0.0148 (4)	0.0132 (4)	0.0148 (4)	0.0049 (4)	0.0032 (4)	0.0021 (4)
O1	0.0351 (17)	0.0227 (15)	0.0163 (14)	0.0150 (13)	0.0074 (13)	0.0052 (12)
O2	0.0177 (14)	0.0232 (15)	0.0453 (19)	0.0067 (12)	0.0157 (14)	0.0028 (14)
O3	0.0320 (16)	0.0164 (14)	0.0256 (15)	0.0124 (13)	0.0133 (13)	0.0082 (12)
O4	0.0158 (13)	0.0201 (14)	0.0213 (15)	0.0030 (12)	0.0036 (12)	−0.0055 (11)
O5	0.0182 (14)	0.0174 (14)	0.0173 (14)	0.0036 (11)	0.0036 (12)	0.0004 (11)
O6	0.0295 (16)	0.0180 (15)	0.0332 (18)	0.0114 (13)	0.0037 (14)	0.0064 (13)
O7	0.0165 (14)	0.0273 (15)	0.0192 (14)	0.0085 (12)	0.0073 (11)	0.0066 (12)
O8	0.0239 (14)	0.0275 (15)	0.0170 (14)	0.0131 (13)	0.0092 (12)	0.0045 (12)
N1	0.0187 (16)	0.0204 (17)	0.0201 (17)	0.0082 (14)	0.0082 (14)	0.0073 (14)
N2	0.0273 (18)	0.0250 (18)	0.0211 (17)	0.0173 (15)	0.0134 (15)	0.0102 (14)
N3	0.0195 (16)	0.0214 (17)	0.0191 (17)	0.0099 (14)	0.0084 (14)	0.0073 (14)
N4	0.0171 (16)	0.0174 (16)	0.0188 (17)	0.0083 (13)	0.0093 (14)	0.0052 (13)
N5	0.0304 (19)	0.0205 (18)	0.0229 (18)	0.0129 (16)	0.0116 (15)	0.0061 (15)
N6	0.0189 (17)	0.0236 (17)	0.0243 (18)	0.0103 (14)	0.0092 (14)	0.0038 (14)
C1	0.031 (2)	0.026 (2)	0.033 (2)	0.0157 (19)	0.0180 (19)	0.0152 (19)
C2	0.031 (2)	0.028 (2)	0.040 (3)	0.0087 (19)	0.018 (2)	0.020 (2)
C3	0.029 (2)	0.052 (3)	0.034 (3)	0.016 (2)	0.020 (2)	0.026 (2)
C4	0.034 (3)	0.047 (3)	0.023 (2)	0.014 (2)	0.015 (2)	0.008 (2)
C5	0.029 (2)	0.029 (2)	0.020 (2)	0.0102 (19)	0.0091 (18)	0.0037 (18)
C6	0.023 (2)	0.029 (2)	0.036 (3)	0.0144 (19)	0.0078 (19)	0.0027 (19)
C7	0.024 (2)	0.052 (3)	0.055 (4)	0.024 (2)	0.001 (2)	−0.004 (3)
C8	0.046 (3)	0.056 (4)	0.061 (4)	0.043 (3)	0.005 (3)	0.007 (3)
C9	0.053 (3)	0.039 (3)	0.058 (4)	0.037 (3)	0.017 (3)	0.012 (3)
C10	0.036 (2)	0.028 (2)	0.029 (2)	0.022 (2)	0.010 (2)	0.0090 (19)
C11	0.031 (2)	0.022 (2)	0.026 (2)	0.0147 (18)	0.0138 (18)	0.0113 (17)
C12	0.033 (2)	0.032 (2)	0.041 (3)	0.018 (2)	0.022 (2)	0.023 (2)
C13	0.032 (2)	0.049 (3)	0.032 (3)	0.019 (2)	0.020 (2)	0.025 (2)
C14	0.030 (2)	0.043 (3)	0.025 (2)	0.012 (2)	0.015 (2)	0.006 (2)
C15	0.025 (2)	0.026 (2)	0.029 (2)	0.0082 (18)	0.0159 (19)	0.0061 (18)
C16	0.023 (2)	0.026 (2)	0.047 (3)	0.0133 (19)	0.008 (2)	−0.001 (2)
C17	0.024 (2)	0.043 (3)	0.055 (3)	0.021 (2)	0.000 (2)	−0.001 (3)
C18	0.042 (3)	0.045 (3)	0.060 (4)	0.035 (3)	0.018 (3)	0.017 (3)
C19	0.038 (3)	0.028 (2)	0.064 (4)	0.022 (2)	0.016 (3)	0.013 (2)
C20	0.024 (2)	0.024 (2)	0.037 (2)	0.0139 (18)	0.0080 (19)	0.0077 (19)
C21	0.035 (3)	0.036 (3)	0.051 (3)	0.020 (2)	0.017 (2)	0.024 (2)
C22	0.057 (3)	0.048 (3)	0.069 (4)	0.041 (3)	0.041 (3)	0.037 (3)
C23	0.079 (4)	0.030 (3)	0.058 (4)	0.035 (3)	0.045 (3)	0.023 (3)
C24	0.055 (3)	0.025 (2)	0.045 (3)	0.011 (2)	0.020 (3)	0.010 (2)
C25	0.038 (3)	0.021 (2)	0.032 (2)	0.011 (2)	0.010 (2)	0.0063 (19)
C26	0.024 (2)	0.035 (2)	0.030 (2)	0.013 (2)	0.0077 (19)	0.004 (2)
C27	0.020 (2)	0.041 (3)	0.039 (3)	0.012 (2)	0.003 (2)	0.004 (2)
C28	0.023 (2)	0.044 (3)	0.051 (3)	0.020 (2)	0.019 (2)	0.020 (2)
C29	0.038 (3)	0.060 (3)	0.046 (3)	0.031 (3)	0.025 (2)	0.013 (3)
C30	0.021 (2)	0.044 (3)	0.030 (2)	0.017 (2)	0.0062 (18)	0.004 (2)

### (2) Geometric parameters (Å, º)

**Table:**

Co1—O4	2.070 (3)	C6—C7	1.376 (7)
Co1—O5	2.080 (3)	C6—H6A	0.9500
Co1—N2	2.179 (3)	C7—C8	1.377 (9)
Co1—N1	2.180 (3)	C7—H7A	0.9500
Co1—N4	2.183 (3)	C8—C9	1.356 (9)
Co1—N3	2.185 (3)	C8—H8A	0.9500
Co2—N6	2.104 (3)	C9—C10	1.398 (6)
Co2—O7	2.113 (3)	C9—H9A	0.9500
Co2—N5	2.123 (3)	C10—H10A	0.9500
Co2—O3^i^	2.141 (3)	C11—C12	1.385 (6)
Co2—O1^i^	2.185 (3)	C11—H11A	0.9500
Co2—O8	2.208 (3)	C12—C13	1.375 (7)
Co2—S1^i^	2.7061 (11)	C12—H12A	0.9500
Co2—S2	2.7107 (10)	C13—C14	1.370 (7)
S1—O2	1.450 (3)	C13—H13A	0.9500
S1—O4	1.476 (3)	C14—C15	1.378 (7)
S1—O1	1.483 (3)	C14—H14A	0.9500
S1—O3	1.493 (3)	C15—H15A	0.9500
S1—Co2^ii^	2.7062 (11)	C16—C17	1.384 (7)
S2—O6	1.451 (3)	C16—H16A	0.9500
S2—O5	1.477 (3)	C17—C18	1.379 (8)
S2—O8	1.488 (3)	C17—H17A	0.9500
S2—O7	1.500 (3)	C18—C19	1.372 (8)
O1—Co2^ii^	2.185 (3)	C18—H18A	0.9500
O3—Co2^ii^	2.141 (3)	C19—C20	1.383 (6)
N1—C5	1.337 (6)	C19—H19A	0.9500
N1—C1	1.337 (6)	C20—H20A	0.9500
N2—C10	1.333 (6)	C21—C22	1.383 (7)
N2—C6	1.355 (6)	C21—H21A	0.9500
N3—C15	1.340 (5)	C22—C23	1.364 (9)
N3—C11	1.344 (5)	C22—H22A	0.9500
N4—C16	1.339 (6)	C23—C24	1.383 (9)
N4—C20	1.340 (6)	C23—H23A	0.9500
N5—C25	1.331 (6)	C24—C25	1.379 (7)
N5—C21	1.344 (6)	C24—H24A	0.9500
N6—C30	1.331 (6)	C25—H25A	0.9500
N6—C26	1.340 (6)	C26—C27	1.381 (7)
C1—C2	1.388 (6)	C26—H26A	0.9500
C1—H1A	0.9500	C27—C28	1.380 (8)
C2—C3	1.384 (8)	C27—H27A	0.9500
C2—H2A	0.9500	C28—C29	1.378 (8)
C3—C4	1.395 (8)	C28—H28A	0.9500
C3—H3A	0.9500	C29—C30	1.393 (7)
C4—C5	1.378 (7)	C29—H29A	0.9500
C4—H4A	0.9500	C30—H30A	0.9500
C5—H5A	0.9500		
			
O4—Co1—O5	173.43 (12)	C2—C1—H1A	118.3
O4—Co1—N2	85.60 (13)	C3—C2—C1	118.7 (4)
O5—Co1—N2	87.84 (12)	C3—C2—H2A	120.6
O4—Co1—N1	89.17 (13)	C1—C2—H2A	120.6
O5—Co1—N1	90.86 (12)	C2—C3—C4	117.9 (4)
N2—Co1—N1	87.48 (13)	C2—C3—H3A	121.0
O4—Co1—N4	96.45 (12)	C4—C3—H3A	121.0
O5—Co1—N4	90.12 (12)	C5—C4—C3	119.4 (5)
N2—Co1—N4	177.57 (14)	C5—C4—H4A	120.3
N1—Co1—N4	91.24 (12)	C3—C4—H4A	120.3
O4—Co1—N3	91.19 (13)	N1—C5—C4	122.9 (4)
O5—Co1—N3	88.85 (12)	N1—C5—H5A	118.5
N2—Co1—N3	93.18 (12)	C4—C5—H5A	118.5
N1—Co1—N3	179.27 (14)	N2—C6—C7	122.5 (5)
N4—Co1—N3	88.08 (12)	N2—C6—H6A	118.8
N6—Co2—O7	92.49 (12)	C7—C6—H6A	118.8
N6—Co2—N5	93.63 (13)	C6—C7—C8	119.6 (5)
O7—Co2—N5	98.33 (13)	C6—C7—H7A	120.2
N6—Co2—O3^i^	96.90 (13)	C8—C7—H7A	120.2
O7—Co2—O3^i^	162.62 (11)	C9—C8—C7	118.4 (5)
N5—Co2—O3^i^	95.64 (12)	C9—C8—H8A	120.8
N6—Co2—O1^i^	93.88 (13)	C7—C8—H8A	120.8
O7—Co2—O1^i^	98.98 (11)	C8—C9—C10	119.8 (5)
N5—Co2—O1^i^	160.79 (12)	C8—C9—H9A	120.1
O3^i^—Co2—O1^i^	65.90 (10)	C10—C9—H9A	120.1
N6—Co2—O8	158.65 (12)	N2—C10—C9	122.3 (5)
O7—Co2—O8	66.37 (10)	N2—C10—H10A	118.8
N5—Co2—O8	92.48 (13)	C9—C10—H10A	118.8
O3^i^—Co2—O8	102.84 (11)	N3—C11—C12	123.2 (4)
O1^i^—Co2—O8	86.87 (11)	N3—C11—H11A	118.4
N6—Co2—S1^i^	101.38 (10)	C12—C11—H11A	118.4
O7—Co2—S1^i^	130.13 (8)	C13—C12—C11	118.9 (4)
N5—Co2—S1^i^	127.73 (10)	C13—C12—H12A	120.6
O3^i^—Co2—S1^i^	33.37 (7)	C11—C12—H12A	120.6
O1^i^—Co2—S1^i^	33.18 (8)	C14—C13—C12	118.5 (4)
O8—Co2—S1^i^	90.91 (8)	C14—C13—H13A	120.8
N6—Co2—S2	125.39 (10)	C12—C13—H13A	120.8
O7—Co2—S2	33.41 (8)	C13—C14—C15	119.6 (5)
N5—Co2—S2	99.84 (10)	C13—C14—H14A	120.2
O3^i^—Co2—S2	133.35 (8)	C15—C14—H14A	120.2
O1^i^—Co2—S2	90.12 (8)	N3—C15—C14	123.0 (4)
O8—Co2—S2	33.27 (7)	N3—C15—H15A	118.5
S1^i^—Co2—S2	110.52 (3)	C14—C15—H15A	118.5
O2—S1—O4	110.31 (18)	N4—C16—C17	123.2 (4)
O2—S1—O1	112.58 (19)	N4—C16—H16A	118.4
O4—S1—O1	109.17 (18)	C17—C16—H16A	118.4
O2—S1—O3	111.06 (18)	C18—C17—C16	119.4 (5)
O4—S1—O3	109.08 (18)	C18—C17—H17A	120.3
O1—S1—O3	104.45 (16)	C16—C17—H17A	120.3
O2—S1—Co2^ii^	117.43 (14)	C19—C18—C17	117.9 (4)
O4—S1—Co2^ii^	132.24 (12)	C19—C18—H18A	121.1
O1—S1—Co2^ii^	53.72 (11)	C17—C18—H18A	121.1
O3—S1—Co2^ii^	52.05 (12)	C18—C19—C20	119.5 (5)
O6—S2—O5	110.02 (17)	C18—C19—H19A	120.2
O6—S2—O8	112.20 (18)	C20—C19—H19A	120.2
O5—S2—O8	109.12 (17)	N4—C20—C19	123.2 (4)
O6—S2—O7	111.68 (18)	N4—C20—H20A	118.4
O5—S2—O7	108.93 (17)	C19—C20—H20A	118.4
O8—S2—O7	104.72 (17)	N5—C21—C22	123.3 (5)
O6—S2—Co2	120.94 (13)	N5—C21—H21A	118.4
O5—S2—Co2	128.99 (12)	C22—C21—H21A	118.4
O8—S2—Co2	54.46 (11)	C23—C22—C21	119.1 (5)
O7—S2—Co2	50.87 (11)	C23—C22—H22A	120.5
S1—O1—Co2^ii^	93.10 (14)	C21—C22—H22A	120.5
S1—O3—Co2^ii^	94.57 (14)	C22—C23—C24	118.2 (4)
S1—O4—Co1	159.38 (19)	C22—C23—H23A	120.9
S2—O5—Co1	169.67 (19)	C24—C23—H23A	120.9
S2—O7—Co2	95.72 (15)	C25—C24—C23	119.5 (5)
S2—O8—Co2	92.27 (14)	C25—C24—H24A	120.3
C5—N1—C1	117.6 (4)	C23—C24—H24A	120.3
C5—N1—Co1	119.9 (3)	N5—C25—C24	123.0 (5)
C1—N1—Co1	122.6 (3)	N5—C25—H25A	118.5
C10—N2—C6	117.3 (4)	C24—C25—H25A	118.5
C10—N2—Co1	122.1 (3)	N6—C26—C27	122.9 (5)
C6—N2—Co1	120.6 (3)	N6—C26—H26A	118.5
C15—N3—C11	116.8 (4)	C27—C26—H26A	118.5
C15—N3—Co1	119.0 (3)	C28—C27—C26	119.1 (4)
C11—N3—Co1	124.2 (3)	C28—C27—H27A	120.4
C16—N4—C20	116.7 (4)	C26—C27—H27A	120.4
C16—N4—Co1	121.3 (3)	C29—C28—C27	118.7 (4)
C20—N4—Co1	121.9 (3)	C29—C28—H28A	120.7
C25—N5—C21	117.0 (4)	C27—C28—H28A	120.7
C25—N5—Co2	122.4 (3)	C28—C29—C30	118.5 (5)
C21—N5—Co2	120.4 (3)	C28—C29—H29A	120.7
C30—N6—C26	117.5 (4)	C30—C29—H29A	120.7
C30—N6—Co2	119.9 (3)	N6—C30—C29	123.2 (4)
C26—N6—Co2	122.3 (3)	N6—C30—H30A	118.4
N1—C1—C2	123.4 (4)	C29—C30—H30A	118.4
N1—C1—H1A	118.3		
			
O2—S1—O1—Co2^ii^	108.27 (16)	Co1—N2—C10—C9	178.3 (4)
O4—S1—O1—Co2^ii^	−128.88 (15)	C8—C9—C10—N2	0.4 (9)
O3—S1—O1—Co2^ii^	−12.33 (17)	C15—N3—C11—C12	1.7 (6)
O2—S1—O3—Co2^ii^	−109.00 (18)	Co1—N3—C11—C12	−179.2 (3)
O4—S1—O3—Co2^ii^	129.22 (15)	N3—C11—C12—C13	−1.4 (7)
O1—S1—O3—Co2^ii^	12.61 (17)	C11—C12—C13—C14	−0.4 (7)
O2—S1—O4—Co1	1.7 (7)	C12—C13—C14—C15	1.6 (7)
O1—S1—O4—Co1	−122.5 (6)	C11—N3—C15—C14	−0.3 (6)
O3—S1—O4—Co1	124.0 (6)	Co1—N3—C15—C14	−179.5 (4)
Co2^ii^—S1—O4—Co1	179.6 (5)	C13—C14—C15—N3	−1.4 (7)
O6—S2—O5—Co1	−43.6 (11)	C20—N4—C16—C17	−1.2 (8)
O8—S2—O5—Co1	−167.1 (11)	Co1—N4—C16—C17	175.5 (4)
O7—S2—O5—Co1	79.2 (11)	N4—C16—C17—C18	0.4 (9)
Co2—S2—O5—Co1	133.7 (10)	C16—C17—C18—C19	1.7 (9)
O6—S2—O7—Co2	−112.94 (17)	C17—C18—C19—C20	−2.8 (9)
O5—S2—O7—Co2	125.34 (15)	C16—N4—C20—C19	0.0 (7)
O8—S2—O7—Co2	8.72 (18)	Co1—N4—C20—C19	−176.7 (4)
O6—S2—O8—Co2	113.01 (16)	C18—C19—C20—N4	2.0 (8)
O5—S2—O8—Co2	−124.80 (14)	C25—N5—C21—C22	−0.8 (8)
O7—S2—O8—Co2	−8.31 (17)	Co2—N5—C21—C22	174.2 (4)
C5—N1—C1—C2	−1.2 (6)	N5—C21—C22—C23	−0.5 (8)
Co1—N1—C1—C2	180.0 (3)	C21—C22—C23—C24	1.8 (8)
N1—C1—C2—C3	0.4 (7)	C22—C23—C24—C25	−1.9 (8)
C1—C2—C3—C4	1.2 (7)	C21—N5—C25—C24	0.7 (7)
C2—C3—C4—C5	−2.1 (7)	Co2—N5—C25—C24	−174.2 (4)
C1—N1—C5—C4	0.3 (7)	C23—C24—C25—N5	0.6 (8)
Co1—N1—C5—C4	179.2 (4)	C30—N6—C26—C27	2.0 (7)
C3—C4—C5—N1	1.3 (7)	Co2—N6—C26—C27	−171.6 (4)
C10—N2—C6—C7	−0.5 (7)	N6—C26—C27—C28	−0.1 (8)
Co1—N2—C6—C7	−178.9 (4)	C26—C27—C28—C29	−1.8 (8)
N2—C6—C7—C8	0.8 (9)	C27—C28—C29—C30	1.8 (8)
C6—C7—C8—C9	−0.5 (10)	C26—N6—C30—C29	−2.1 (7)
C7—C8—C9—C10	−0.1 (10)	Co2—N6—C30—C29	171.7 (4)
C6—N2—C10—C9	−0.1 (7)	C28—C29—C30—N6	0.2 (8)

Symmetry codes: (i) *x*−1, *y*−1, *z*−1; (ii) *x*+1, *y*+1, *z*+1.

### (2) Hydrogen-bond geometry (Å, º)

**Table:**

*D*—H···*A*	*D*—H	H···*A*	*D*···*A*	*D*—H···*A*
C1—H1*A*···O6	0.95	2.63	3.563 (6)	167
C2—H2*A*···O2^iii^	0.95	2.52	3.219 (5)	131
C5—H5*A*···O4	0.95	2.42	3.009 (5)	120
C6—H6*A*···O5	0.95	2.56	3.054 (5)	112
C6—H6*A*···O7	0.95	2.47	3.322 (6)	149
C10—H10*A*···O4	0.95	2.53	3.010 (5)	112
C12—H12*A*···O7^iv^	0.95	2.60	3.271 (5)	128
C15—H15*A*···O5	0.95	2.45	3.017 (5)	118
C16—H16*A*···O2	0.95	2.19	3.139 (6)	176
C20—H20*A*···O5	0.95	2.51	3.091 (5)	119
C20—H20*A*···O6	0.95	2.32	3.272 (5)	175
C25—H25*A*···O8	0.95	2.55	3.162 (5)	123
C30—H30*A*···O7	0.95	2.54	3.116 (5)	119

Symmetry codes: (iii) *x*−1, *y*−1, *z*; (iv) *x*+1, *y*+1, *z*.
